# Fast-response self-powered double-heterojunction n-ZnO/p-ZnTe/n-Si photodetector†

**DOI:** 10.1039/d5na00331h

**Published:** 2025-06-11

**Authors:** Ethar Yahya Salih, Mohamed Hassan Eisa, Mustafa K. A. Mohammed, Asmiet Ramizy, Osamah Aldaghri, Raid A. Ismail, Khalid Hassan Ibnaouf

**Affiliations:** a College of Energy and Environmental Sciences, Al-Karkh University of Science Baghdad 10081 Iraq ethar988@gmail.com ethar@kus.edu.iq; b Department of Physics, College of Science, Imam Mohammad Ibn Saud Islamic University (IMSIU) Riyadh 13318 Saudi Arabia; c College of Remote Sensing and Geophysics, Al-Karkh University of Science Baghdad 10011 Iraq; d College of Science, University of Anbar Anbar 31001 Iraq; e Applied Science Department, University of Technology Baghdad 10066 Iraq

## Abstract

This study elucidates a novel, fast-response, self-driven double-heterojunction (n-ZnO/p-ZnTe/n-Si) photodetector fabricated *via* the rapid pulsed laser deposition (PLD) technique. The proposed geometry exhibits dual-responsive behavior under ultraviolet (375 nm) and visible (530 nm) incident wavelengths due to the heterojunctions (n-ZnO/p-ZnTe/Si and p-ZnTe/n-Si). Under a 0.5 bias condition, the former exhibited photo-responsivity (*R*_λ_) and photo-detectivity (D*) of 64.03 mA W^−1^ and 5.19 × 10^14^ Jones at 375 nm, while the latter demonstrated values of 53.20 mA W^−1^ and 2.44 × 10^14^ Jones at 530 nm, respectively; lower figure-of-merits were observed at higher and/or lower wavelengths. However, a higher applied bias contributes to a significant *R*_λ_ and D* augmentation under these wavelengths. The observed characteristics were found to decrease at high incident light intensity, which suggests a negative correlation between the calculated parameters, with an *R*^2^ value close to unity (*R*^2^ = −1). At zero applied bias, the proposed system demonstrated a stable performance over a period of 5 days with less than 1.5% variation. The response/recovery times for the proposed heterojunctions were 88/90 ms and 89/94 ms under 375 nm and 530 nm, respectively.

## Abstract 2

A double-heterojunction self-biased fast-response ZnO/ZnTe/Si-based photodetector was proposed for multi-band wavelength detection applications.

## Graphical abstract

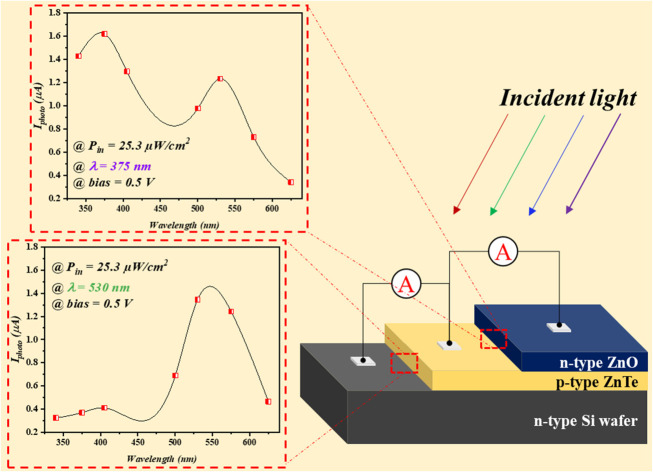

## 1. Introduction

To date, photodetector-based broadband wavelength detection, which spans a wide range from ultraviolet (UV) to near-infrared (NIR), is being extensively explored due to its wide-reaching applications, including remote control, environmental monitoring, and optical communications.^1–4^ In this context, there are two main photo-detection working mechanisms: photo-conductive and photo-voltaic modes.^5–7^ The former delivers comparatively high multi-cycled carrier phenomenon-based photo-gain; it usually, but not unavoidably, suffers from a slow recombination rate, mainly attributed to trapped carriers. The latter, however, offers faster response/recovery rates as a result of the generated built-in electric field within the formed junction; this, in turn, promotes the separation and recombination of electron/hole pairs where an external bias is not required, bringing about a reduced recombination rate, along with an enhanced response speed. This includes mono/hetero junctions as well as Schottky barrier configurations.^8–10^ Recently, heterostructure-based silicon (Si) photodetectors have been reported to be efficient compared to their counterparts, including homo and Schottky-structured photodetectors. Among these, double-heterojunction photodetectors demonstrated a number of upsides, such as broadband detection phenomenon, relatively fast response/recovery times, as well as pronounced figure-of-merits at zero and/or extremely low external potential, due to which low optical signal can be detected remotely without the need for energy consumption.^11–13^ In this context, several semiconductor-based photodetectors have been reported, including CuO-TiO_2_/Si, SnO_2_/CuO/Si, and CuO/Cu_2_O/Si;^14–16^ such geometries exhibited relatively sound figure-of-merits along fast response behavior. Further, 2D materials were also considered for such designs, such as WSe_2_/WS_2_/Si and PtSe_2_/GaAs/Si;^17,18^ the latter exhibited higher photoresponsive performance in the device as compared to the former. From this perspective, we report a novel, fast-response, self-powered double-heterojunction (n–p and p–n) photodetector (ZnO/ZnTe/Si) fabricated using a rapid pulsed laser deposition (PLD) approach. ZnO is a promising II–VI semiconductor for various opto-electronic applications, exhibiting considerable lasing and photoluminescence at room temperature.^19^ In addition, ZnO exhibits n-type conductivity characteristics, a direct/wide optical band gap of ∼3.3 eV, and a large exciton binding energy (60 meV), allowing UV light absorption.^20–22^ ZnTe, with p-type conductivity, is a semiconductor compound (II–VI), with attractive opto-electronic characteristics, enabling its tunability for various light-based applications. ZnTe has a direct optical bandgap (∼2.23 eV) with a relatively high absorption coefficient, which allows the absorption of visible light.^23–25^ Herein, comprehensive photoresponsive characteristics of the fabricated device (n-ZnO/p-ZnTe/n-Si) are demonstrated, considering both incident wavelength and intensity profiles. Furthermore, detailed time-resolved characteristics are also elucidated. Based on the reported data, the double-heterojunction photodetector contributes to improved carrier separation and transport as a result of the electric fields generated in the n-ZnO/p-ZnTe/Si and p-ZnTe/n-Si heterojunctions, which, in turn, reduce recombination and enhance photo-responsive characteristics. Furthermore, the wide-bandgap ZnO layer serves as a blocking layer, lowering noise current and improving signal-to-noise ratio, resulting in increased responsivity and detectivity of the photodetector.

## 2. Experimental section

**Fig. 1:**
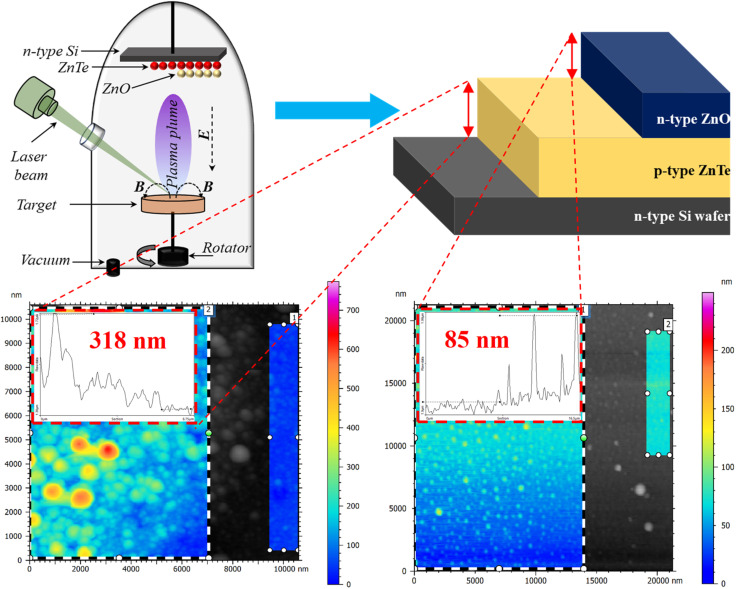
Schematic of the fabrication method and the proposed geometry; the inset shows the thickness profile obtained using the AFM technique.

A single-side polished n-type Si wafer (Sigma-Aldrich, 99.99%, 111, 500 μm) was subjected to a multi-cycle cleaning process. Subsequently, ZnTe (Sigma-Aldrich, 99.99%) was deposited on the Si wafer through the PLD technique under a vacuum pressure of 10^−4^ mbar; this was achieved using 500 laser pulses at a fluence of 6.37 J cm^−2^. The utilized ZnTe target, acquired after 5 tons of mechanical press, was placed within the PLD chamber prior to the vacuum process. In detail, the distance between the ZnTe target and the Si wafer was fixed to 5 cm, while the laser utilized was Q-switched Nd:YAG-second harmonic (532 nm); other parameters such as duration of pulse and repetition frequency were adjusted to 10 ns and 6 Hz, respectively; a detailed PLD illustration is shown in Fig. 1 (upper, left-side). In a similar deposition process, ZnO (Grafi Technology, 99.9%) was also deposited on top of the pre-deposited ZnTe layer, considering similar irradiation environments. However, different layer sizes were considered, allowing for Ag ohmic contacts (Fig. 1, upper-right side). The addressed contacts were acquired for complete device design *via* the thermal evaporation approach at a vacuum pressure of 1 × 10^−6^ mbar attained *via* two steps, rotary and diffusion processes.

The morphological and structural analyses were conducted utilizing field emission scanning electron microscopy (FE-SEM, SU8030, Hitachi) and X-ray diffraction (AXS D8, Bruker), respectively. Atomic force microscopy (AFM, C3000, Flex-Axiom) was used to investigate the thickness profile of the deposited layers (inset in Fig. 1), where ZnO and ZnTe demonstrated thicknesses of 85 nm and 318 nm, respectively. The optical absorption properties of the utilized layers were investigated using ultraviolet-visible light spectroscopy (UV-Vis, UV-3600, Shimadzu). Finally, the optoelectronic characteristics were determined using a source-measure unit (Keithley 2041, SMU) with bandpass optical filters and a peak transmission range of 340–625 nm (Thorlab). Herein, the incident light density was measured using a LX2-illuminance meter (Sanwa, Japan). Moreover, the time-dependent features were projected considering 10% and 90% of the full current gained through a histogram search; the pulse width was set to ∼600 ms, while a long-stability examination was conducted over 5 days.

## 3. Results and discussion

**Fig. 2:**
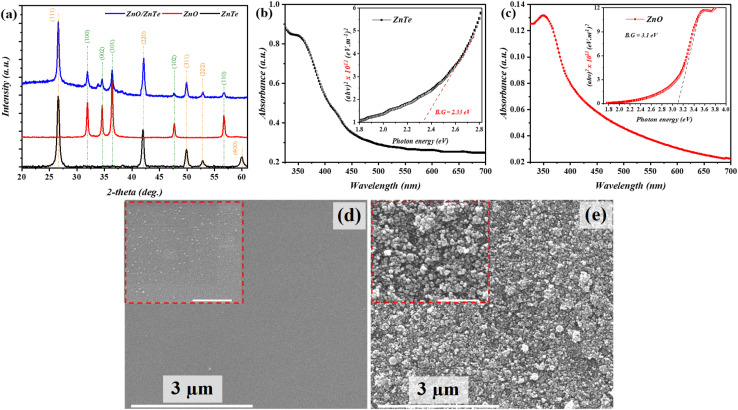
Microstructural analysis of the deposited layers; (a) XRD, (b) (c) UV-Vis of ZnTe and ZnO, (d) and (e) FE-SEM of ZnTe and ZnO, with an inset scale bar of 500 nm.

XRD patterns (Fig. 2a) revealed a successful zinc-blended ZnTe phase (black-line) deposition with peaks located at 2*θ* ≈ 26.60°, 42.02°, 49.93°, 52.85°, and 60.03°; these are related to the *hkl* planes of (111), (220), (311), (222), and (400) respectively, which agrees with JCPDS report #15-0746. Further, the peaks located at 2*θ* ≈ 31.92°, 34.58°, 36.40°, 47.68°, and 56.70° (red-line) corresponded to the successful deposition of the hexagonal ZnO phase structure (JCPDS report #05-0669) along the (100), (002), (101), (102), and (110) planes, respectively. The proposed heterojunction (ZnO/ZnTe) exhibited combined peaks of both deposited structures (blue line), indicating the successful formation of the ZnO/ZnTe heterostructure; however, the (400) peak within the ZnTe profile was not located in the heterostructure. Herein, the optical phenomena of the proposed layers revealed a cut-off phenomenon at around 532 nm and 375 nm for ZnTe and ZnO layer, respectively; the former, however, exhibited a wider absorption spectrum compared to the latter. Additionally, the estimated optical bandgaps using the Tauc relation^26,27^ yielded values of 2.33 eV and 3.1 eV for ZnTe and ZnO (inset of Fig. 2b and c), respectively. The topographies of the deposited ZnTe layer (Fig. 2d) revealed compact and uniformly distributed nanoparticles over a large area with an average nanoparticle diameter of 25.47 nm (inset in Fig. 2(d)). As depicted in Fig. 2(e), the ZnO layer exhibited a compact deposited layer, along with a certain extent of non-uniform nanoparticle distribution on top of the ZnTe layer; the average nanoparticle diameter is estimated to be 30.3 nm, as shown in the inset of Fig. 2(e).

**Fig. 3:**
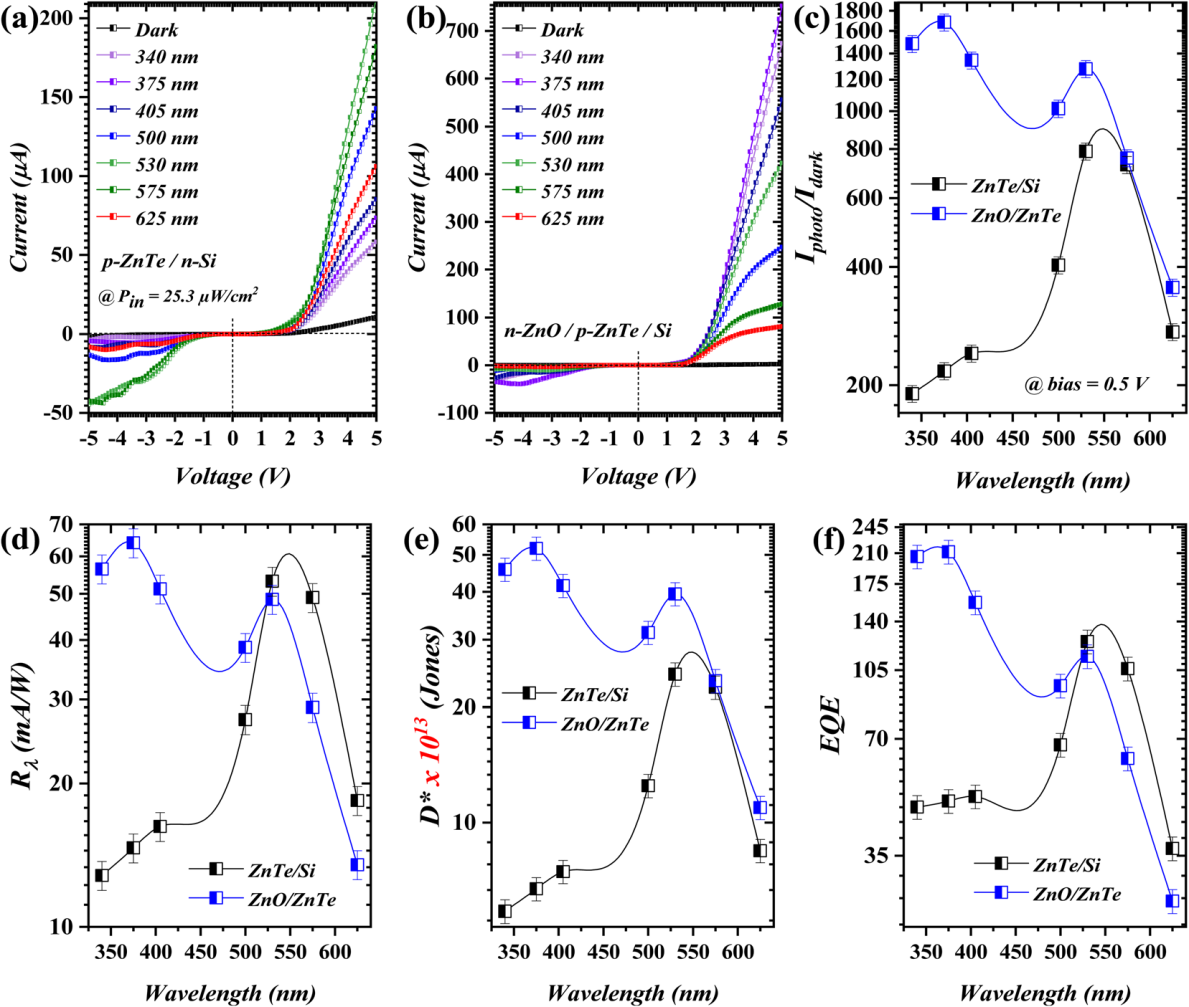
Incident wavelength dependency of *I*–*V* characteristics of (a) p-ZnTe/n-Si and (b) n-ZnO/p-ZnTe/Si, and the figure-of-merits are presented in (c) *I*_photo_/*I*_dark_, (d) *R*_λ_, (e) D*, and (f) EQE.

Fig. 3(a) and (b) illustrate the *I*–*V* characteristics under dark and illumination at different wavelengths of the fabricated double-heterojunction (p-ZnTe/n-Si and n-ZnO/p-ZnTe/Si) photodetector, respectively. In detail, non-linear and unsymmetrical *I*–*V* behavior, in both bias directions, under dark conditions could be observed for both heterojunctions with well-oriented rectification profiles (7.05 and 28.9), respectively; see the ESI (Fig. S2)† for the illustration of the dark *I*–*V* curve. Such a behavior, where *I*_dark_ is lower in the case of n-ZnO/p-ZnTe/Si, could be due to the role of ZnO (with a high CB offset and B. G. = 3.1 eV), acting as an additional junction, which, in turn, limits the charge carriers, particularly in reverse bias.^28^ The related ideality factors (*n*) were found to be 3.63 and 1.7 for p-ZnTe/n-Si and n-ZnO/p-ZnTe/Si, respectively (Fig. S2†); these were calculated according to Cheung's model.^29^ Upon illumination, the current increases significantly in a wavelength-dependent manner under both bias directions; this, in turn, suggests a robust photovoltaic effect. The demonstrated *I*–*V* curves, under illumination conditions, revealed a strong visible light response (Fig. 3a, 530 nm), which was pre-expected from the optical analysis (*λ*_cut-off_ for ZnTe). A higher photo-current (*I*_photo_) could be observed in the n-ZnO/p-ZnTe/Si profile (Fig. 3b), particularly under *λ*_375_ (λ_cut-off_ = 375 nm). Such singularity was further inspected from the *I*_photo_/*I*_dark_ profile (Fig. 3c) where n-ZnO/p-ZnTe/Si exhibited a higher ratio compared to that of p-ZnTe/n-Si. These characteristics were observed at 0.5 V, which further validates the self-driven feature of the proposed double-heterojunction device. The *I*_photo_/*I*_dark_ ratio was found to be 789 and 1682 for p-ZnTe/n-Si and n-ZnO/p-ZnTe/Si. The photo-responsivity [*R*_λ_ = *I*_photo_/*P*_in_],^30^ as shown in Fig. 3(d), exhibits a strong light absorption response under visible wavelengths; such occurrence was also observed in the n-ZnO/p-ZnTe/Si heterojunction. However, the corresponding setting delivered a higher UV absorption trend. As evidenced by the *R*_λ_ profile (Fig. 3d), ZnO contributes to minimizing the rate of charge carrier recombination by reducing the defect/trap states. In detail, ZnO with outstanding n-type conductivity and direct wide bandgap could function as a passivation layer at the ZnO/ZnTe interface, resulting in a lower electron–hole recombination rate.^28^ This was further validated through Urbach energy (EU) calculation-based UV-Vis data (Fig. S1†). The deposited ZnO film exhibited a relatively low EU (621 meV), as determined through the exponential region of the absorption edge, indicating a lower density of defect and tail states in the bandgap. Thus, it is suggested that the reduction of sub-bandgap states implies fewer non-radiative recombination centers, which in turn supports the claim of a low recombination rate. Further, in the case of n-ZnO/p-ZnTe/Si, Si floats without any electrical contact, leading to the so-called photo-gating effect at the bottom of the proposed double-heterojunction.^17^ The peak values of *R*_λ_ were 53.20 mA W^−1^ and 64.03 mA W^−1^ at 530 nm and 375 nm wavelengths for the devices, respectively. This behavior, for heterojunction setting, was found to be decreasing at lower/higher incident wavelengths for the considered geometry. Subsequently, photo-detectivity [D* = *R*_λ_(*A*)^0.5^/(2*qI*_dark_)^0.5^]^31^ indicated the ability of the proposed double-heterojunction to distinguish extremely low incident optical signals with D* values of 2.44 × 10^14^ Jones and 5.19 × 10^14^ Jones under the pronounced wavelengths, respectively. The external quantum efficiency [EQE = 1240 × (*R*_λ_/*λ*)]^32^ revealed similar performance to those of *R*_λ_ and D*, with peak values of 124.4 and 211.7 at wavelengths of 530 nm and 375 nm, respectively. The investigated figure-of-merits exhibited noticeably enhanced trends at 3 V (1.46 A W^−1^ for n-ZnO/p-ZnTe/Si @ 375 nm) with respect to the heterojunction considered; see ESI (Fig. S3).†

**Fig. 4:**
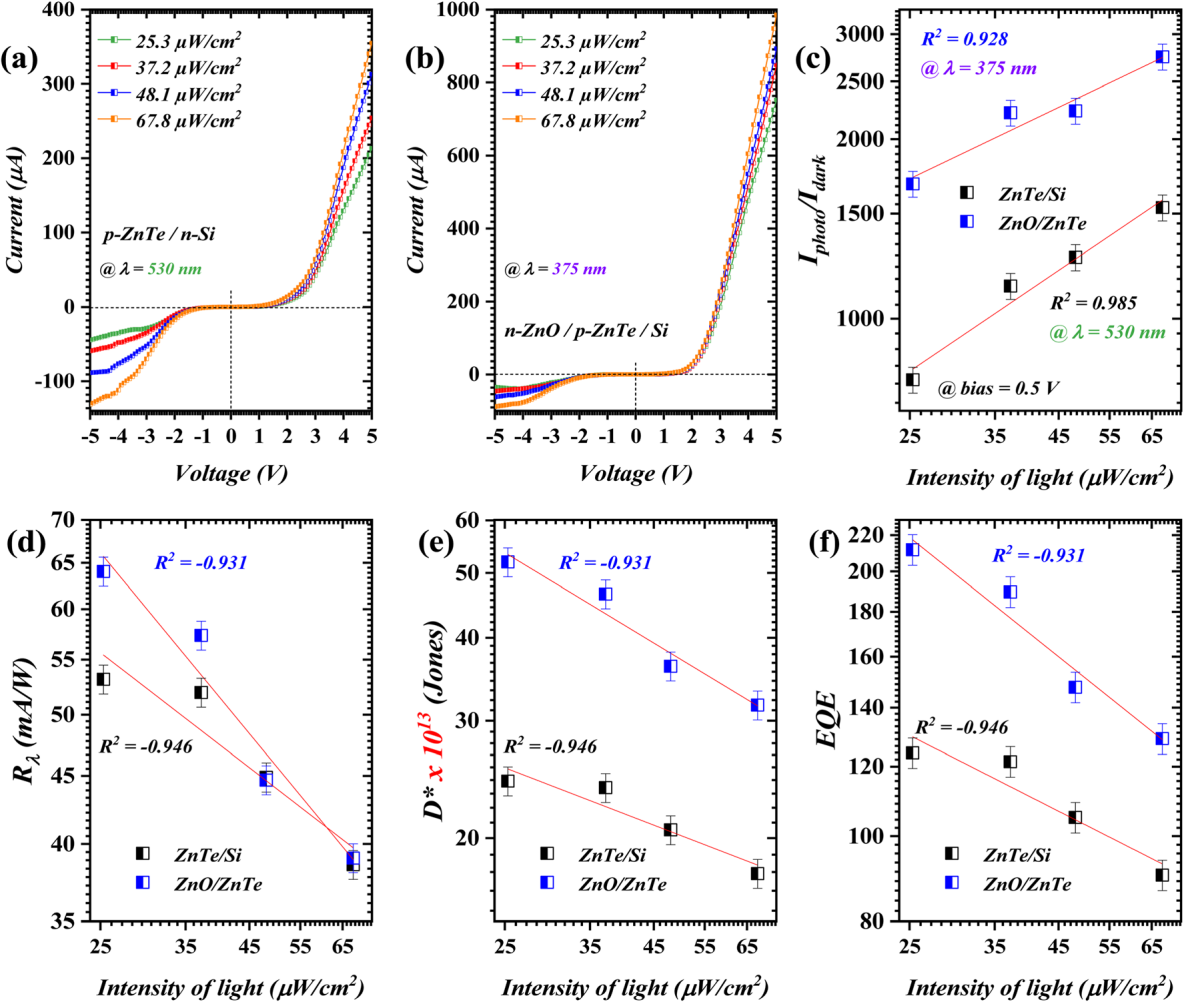
Incident power density dependency of *I*–*V* characteristics of (a) p-ZnTe/n-Si and (b) n-ZnO/p-ZnTe/Si, with figure-of-merits are presented in (c) *I*_photo_/*I*_dark_, (d) *R*_λ_, (e) D*, and (f) EQE.

In terms of the incident light intensity, the proposed double-heterojunction structure revealed a clear intensity-dependent performance, which can be clearly seen in Fig. 4(a and b), at the optimum incident wavelength (*λ*_cut-off_). In detail, the intended double-heterojunction generates a higher rate of electron–hole pairs at a higher light intensity; this allows more photons to be absorbed through the depletion region, resulting in an increased Iphoto^33,34^. This was further authenticated using the Pearson correlation coefficient in the *I*_photo_/*I*_dark_ profile (Fig. 4c). In particular, *R*^2^ close to unity entitles, as a function of light intensity, the ability of a heterojunction to reduce the recombination rate of the photoexcited electrons–holes pairs; the attained *R*^2^ values were 0.985 and 0.928 for p-ZnTe/n-Si and n-ZnO/p-ZnTe/Si, respectively. The addressed profile revealed a higher ratio for the latter heterojunction due to the ability of both ZnO (*λ* ≤ 375 nm) and ZnTe (*λ* ≤ 530 nm) to absorb short/mid wavelengths. The built-in electric field formed at the p-ZnTe/n-Si interface is smaller than that of n-ZnO/p-ZnTe/Si due to the fact that the n-ZnO/p-ZnTe/Si exhibited a small difference in terms of band alignment and work function. The *R*_λ_ demonstrated a decreasing trend with light intensity, which could be due to the inversely proportional relation between the input power and *R*_λ_ (*i.e.*, *R*_λ_ ∝ *P*^−1^). Specifically, values of 38.59 mA W^−1^ and 39.04 mA W^−1^ for ZnTe/n-Si and n-ZnO/p-ZnTe/Si structures at 67.8 μW cm^−2^; a similar behavior was observed at 3 V with a significantly increased profiles (Fig. S4†). In terms of D* and EQE profiles (Fig. 4e and f), the obtained values delivered similar trend to that of *R*_λ_ with *R*^2^ close to unity (*R*^2^ ≅ −1), signifying negative correlation between the addressed figure-of-merits and incident light intensity increment.

**Fig. 5:**
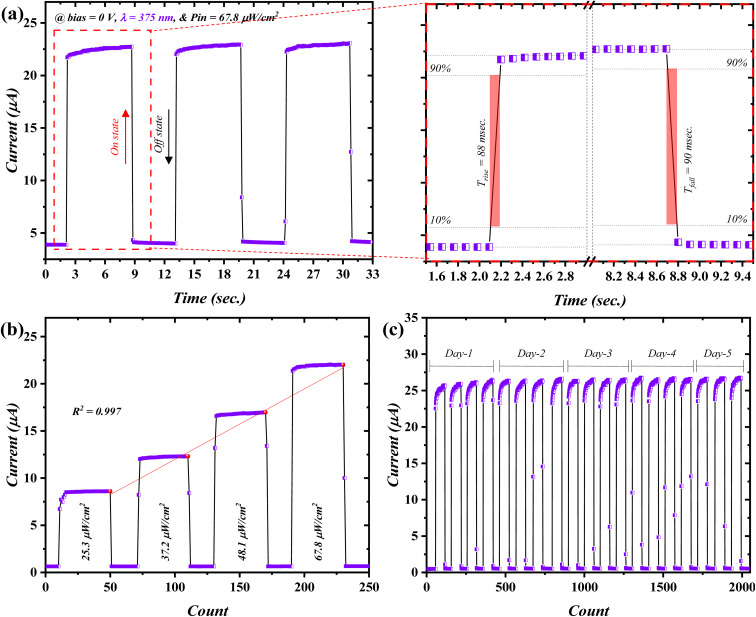
Time-resolved characteristics of ZnO/ZnTe; (a) switching behavior, (b) power-based time-dependent profile, and (c) long-term stability over a period of 5 days.

The response base to incident light of a photodetector is measured through time-resolved characteristics (Fig. 5). The fabricated n-ZnO/p-ZnTe/Si double-heterojunction delivered a relatively fast-response base as a function of the incident wavelength (*λ* = 375 nm), considering zero bias voltage through which a self-powered singularity can be indicated. The photodetector was investigated with three uninterrupted cycles with a pulse width of ∼600 ms (Fig. 5a). The proposed n-ZnO/p-ZnTe/Si geometry revealed a fast response/recovery time profile with a period of 88 ms and 90 ms, respectively, inset of Fig. 5a; this was observed with an *I*_on_/*I*_off_ ratio of 5.96. The attained response time was shorter in comparison to that of recovery, which evidences longer recombination than the separation window of electron/hole pairs.^35^ The time-dependence feature was also evaluated in terms of the incident light intensity (Fig. 5b), where the inspected heterojunction demonstrated a linear current increment as a function of incident light with *R*^2^ = 0.997. Consequently, the stability of the device was tested over a long-term 5-day period (Fig. 5c). The device exhibited stable response/recovery states, indicating both robustness and reproducibility of the device. The p-ZnTe/n-Si heterostructure is presented in Fig. S5† and exhibits a response/recovery of 89/94 ms.

**Fig. 6:**
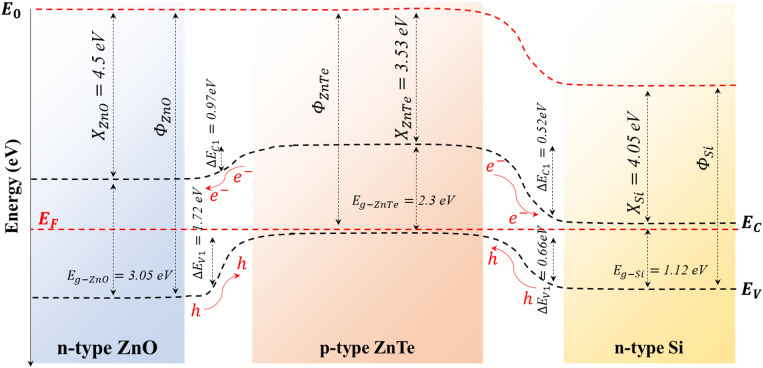
Energy band-diagram alignment of the fabricated double-heterojunction photodetector.

The suggested band diagram of the proposed double-heterojunction is elucidated in Fig. 6. In both n-ZnO/p-ZnTe and p-ZnTe/n-Si setups, where zero bias is considered, a built-in field (*V*_bi_) is acquired because of the difference in energy levels; this occurs in the direction from ZnTe to ZnO and Si. Upon a bias application, the photodetector forms a depletion region along the electric field (*V*_bi_ + *V*_R_) that contributes to the electron–hole separation process. Furthermore, the energy band diagram of the n-ZnO/p-ZnTe/n-Si double heterojunction exhibits efficient carrier separation and transport on account of the band offsets and built-in electric fields at the n-ZnO/p-ZnTe and p-ZnTe/n-Si interfaces. In detail, ZnTe, with a higher conduction band than that of ZnO, allows electron transfer from ZnTe to ZnO; a similar behaviour is observed in the case of p-ZnTe/n-Si. This allows the discontinuity of bands, charge dynamics, and reduced recombination. During the light off-state, an abrupt reduction in the photo-generated carriers forces the device to shut off under reverse bias potential; thus, the current vanishes rapidly. This, in turn, results in a relatively fast response base to various optical singles.^36^

## 4. Conclusion

A novel fast-response self-driven double-heterojunction n-ZnO/p-ZnTe/n-Si photodetector was fabricated using the PLD approach. The fabricated n-ZnO/p-ZnTe and p-ZnTe/n-Si heterojunction devices demonstrated dual-response characteristics at 375 nm and 530 nm, respectively. These devices exhibited *R*_λ_ and D* of 32.02 and 26.58 mA W^−1^ and 5.19 × 10^14^ and 2.44 × 10^14^ Jones at 375 nm and 530 nm, respectively, achieved at an applied bias of 0.5; however, a substantial figure-of-merits increment was attained at 3 V. In terms of the incident wavelength intensity, the investigated parameters revealed reduction profiles at a higher intensity with *R*^2^ = −1. The proposed n-ZnO/p-ZnTe and p-ZnTe/n-Si demonstrate a rather fast response/recovery time of 88/90 and 89/94 ms, respectively. In addition, the stability of the double heterojunction was examined over a period of 5 days, where stable and reproducible behavior were observed.

## Author contributions

E. Y. S., A. R., and R. A. I. conceived the idea of the device and its fabrication process. E. Y. S. and M. K. A. M. performed the device electrical measurements and related data analysis. M. H. E., O. A., and K. H. I. conducted the optical and structural characterizations. All authors contributed equally to the discussion and preparation of the manuscript.

## Conflicts of interest

There are no conflicts to declare.

## Supplementary Material

NA-007-D5NA00331H-s001

## Data Availability

All data are available from the corresponding author upon request.
