# Supplementary material for: Fast-response self-powered double-heterojunction n-ZnO/p-ZnTe/n-Si photodetector
Source: Nanoscale Adv. 2025 Jun 11;7(14):4461–8. doi: 10.1039/d5na00331h (PMC12152967; doi:10.1039/d5na00331h)
Supplement: NA-007-D5NA00331H-s001 [file NA-007-D5NA00331H-s001.pdf]

Supplementary Information (SI) for Nanoscale Advances.
This journal is © The Royal Society of Chemistry 2025

Supplementary Information

Fast-response self-powered double-heterojunction ZnO/ZnTe/Si photodetector

Ethar Yahya Salih [1, *], Mohamed Hassan Eisa [2], Mustafa K. A. Mohammed [3], Asmiet Ramizy [4], Osamah Aldaghri [2], Raid A. Ismail [5], Khalid Hassan Ibnaouf [2]

[1] College of Energy and Environmental Sciences, Al-Karkh University of Science, Baghdad 10081, Iraq

[2] Department of Physics, College of Science, Imam Mohammad Ibn Saud Islamic University (IMSIU), Riyadh, 13318, Saudi Arabia

[3] College of Remote Sensing and Geophysics, Al-Karkh University of Science, Baghdad 10011, Iraq

[4] College of Science, University of Anbar, Anbar 31001, Iraq

[5] Applied Science Department, University of Technology, Baghdad 10066, Iraq

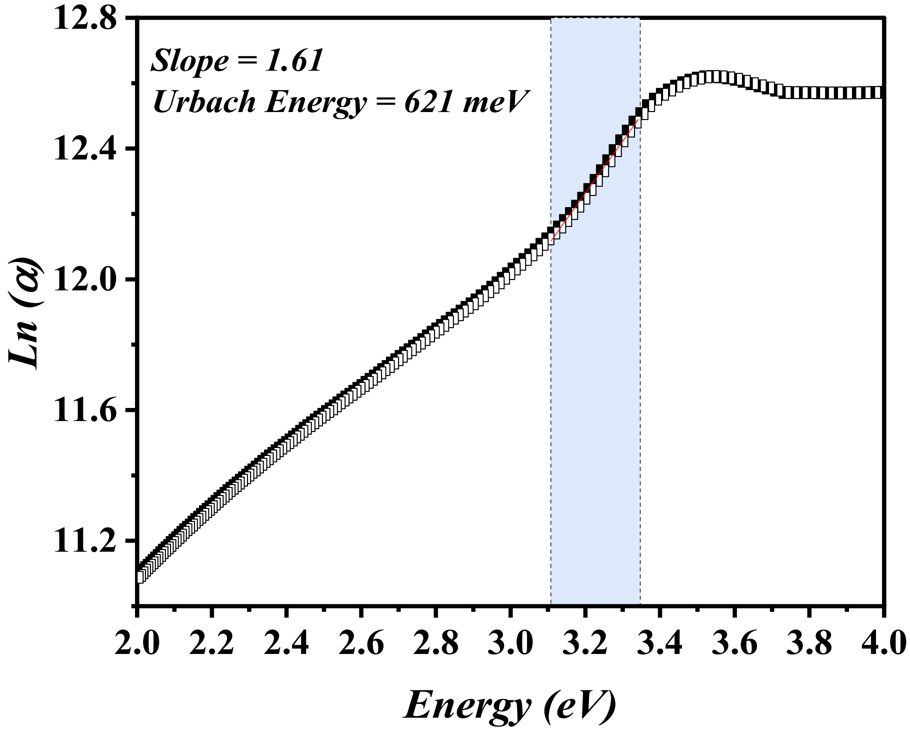

Figure *S1*: Urbach energy analysis of ZnO layer.

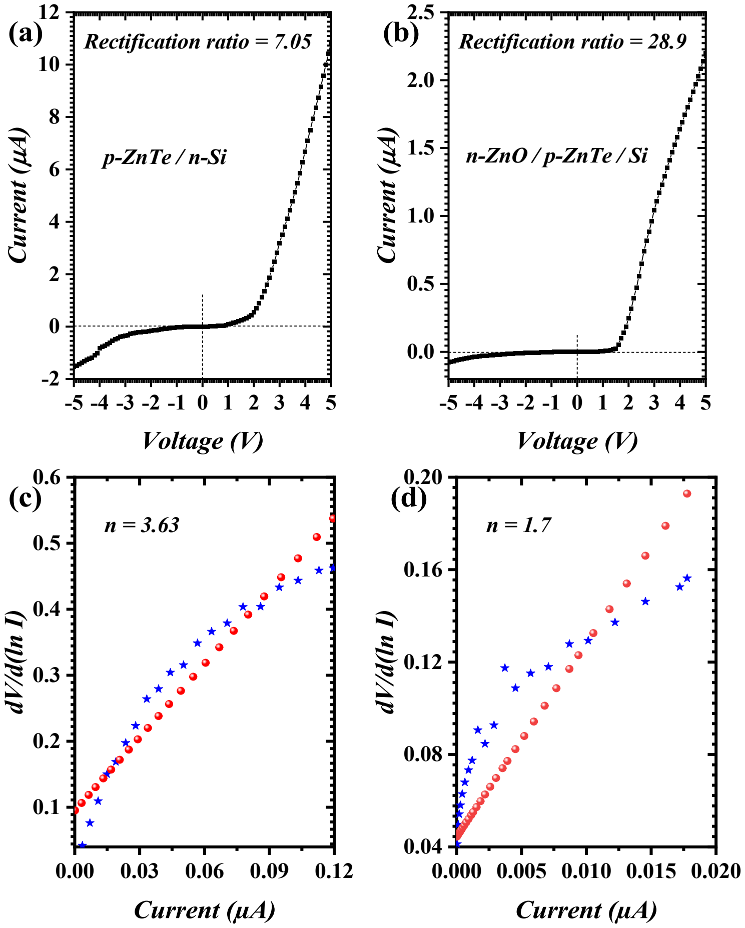

Figure *S2*: I-V characteristics under dark setting (a) ZnO/ZnTe and (b) ZnTe/Si and the related ideality factors of (v) ZnO/ZnTe and (d) ZnTe/Si.

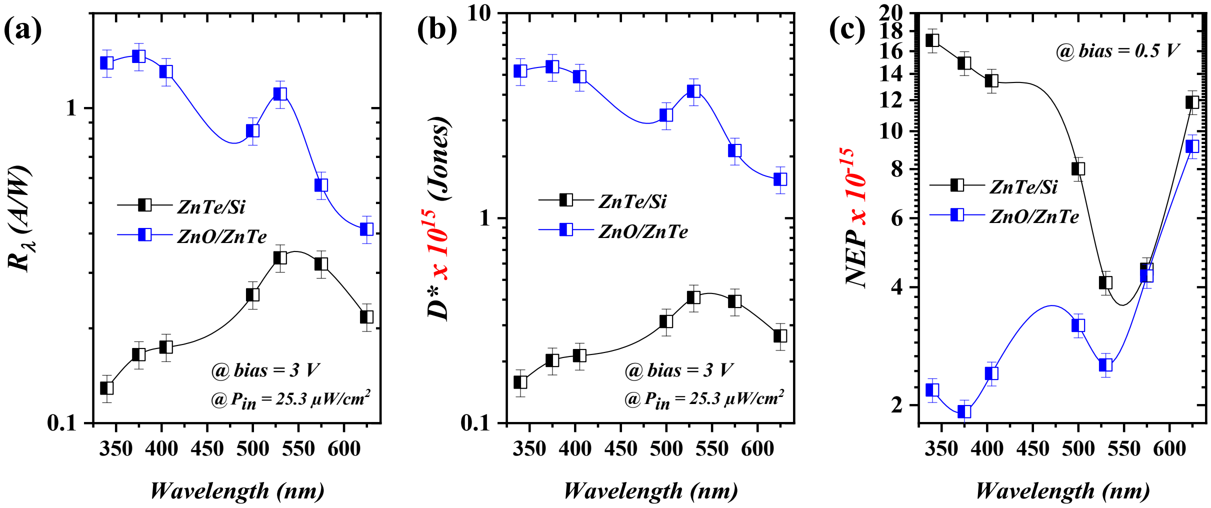

Figure *S3*: Figure-of-merits of the investigated geometries as a function in incident wavelength @ 3 bias voltage; (a) photo-responsivity and (b) photo-detectivity, while (c) NEP at 0.5 bias.

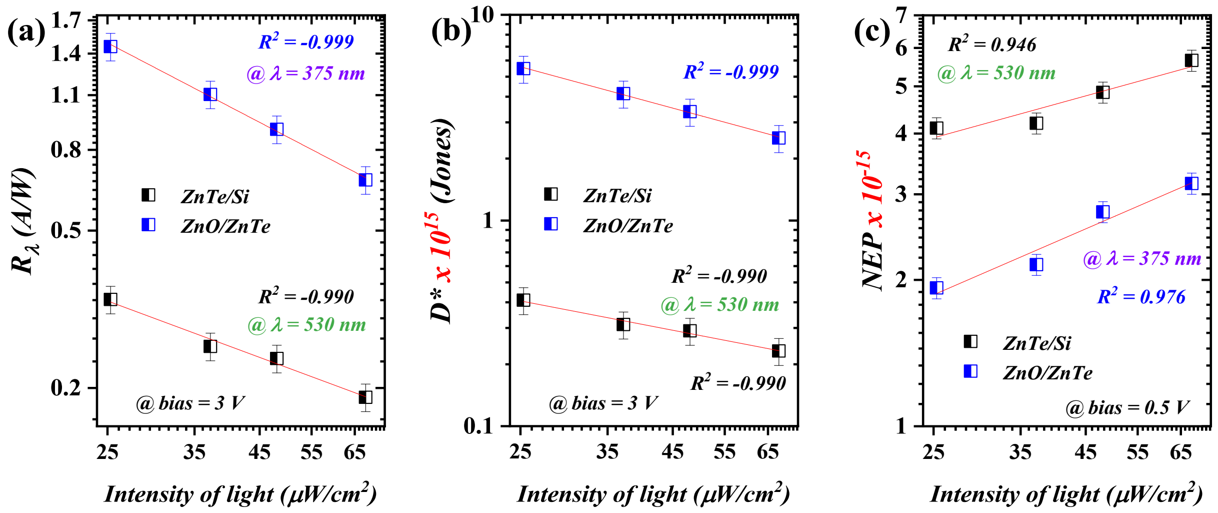

Figure ***S4***: Figure-of-merits of the investigated geometries as a function in incident light intensity @ 3 bias voltage; (a) photo-responsivity and (b) photo-detectivity, while (c) NEP at 0.5 bias.

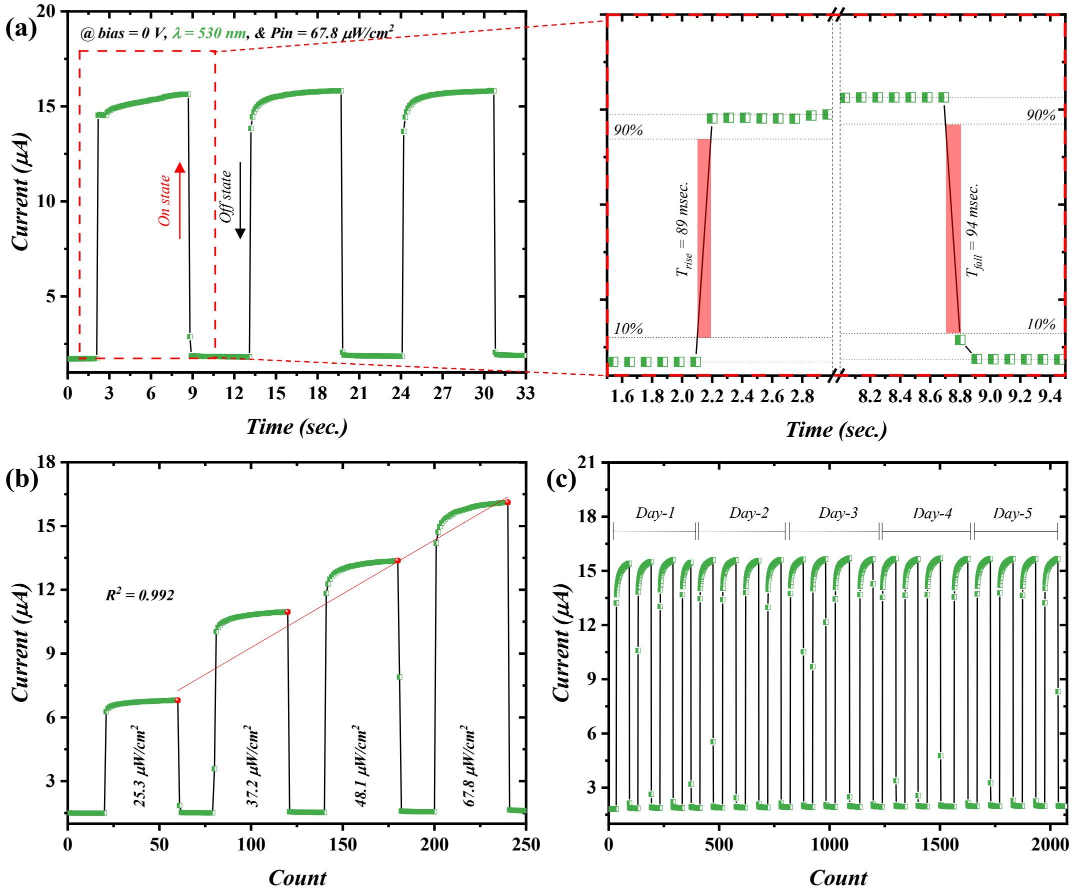

Figure ***S5***: Time-resolved characteristics of ZnTe/Si; (a) switching behavior, (b) power-based time dependent profile, and (c) long-term stability with period of 5 days.
